# The symbiont side of symbiosis: do microbes really benefit?

**DOI:** 10.3389/fmicb.2014.00510

**Published:** 2014-09-26

**Authors:** Justine R. Garcia, Nicole M. Gerardo

**Affiliations:** Gerardo Lab, Department of Biology, O. Wayne Rollins Research Center, Emory University, Atlanta, GAUSA

**Keywords:** mutualism, microbial fitness, host–microbe interactions, symbiont transmission, endosymbiosis

## Abstract

Microbial associations are integral to all eukaryotes. Mutualism, the interaction of two species for the benefit of both, is an important aspect of microbial associations, with evidence that multicellular organisms in particular benefit from microbes. However, the microbe’s perspective has largely been ignored, and it is unknown whether most microbial symbionts benefit from their associations with hosts. It has been presumed that microbial symbionts receive host-derived nutrients or a competition-free environment with reduced predation, but there have been few empirical tests, or even critical assessments, of these assumptions. We evaluate these hypotheses based on available evidence, which indicate reduced competition and predation are not universal benefits for symbionts. Some symbionts do receive nutrients from their host, but this has not always been linked to a corresponding increase in symbiont fitness. We recommend experiments to test symbiont fitness using current experimental systems of symbiosis and detail considerations for other systems. Incorporating symbiont fitness into symbiosis research will provide insight into the evolution of mutualistic interactions and cooperation in general.

## INTRODUCTION

Microbes have been recognized as an important force in eukaryotic evolution (McFall-Ngai et al., 2013), but recognition of the impact of eukaryotes on microbial evolution has lagged behind. Interspecies interactions between microbes and eukaryotic hosts fall on a continuum from parasitism to mutualism. Fitness effects of these interactions are routinely investigated in hosts, but it is necessary to consider both partners to understand how interactions evolve and persist. There is a robust framework for understanding how parasitic interactions promote the fitness of parasitic microbes (pathogens), but the microbe’s perspective has largely been ignored in putatively mutualistic interactions, and it is unknown whether most non-parasitic microbes benefit from host association.

**FIGURE 1:**
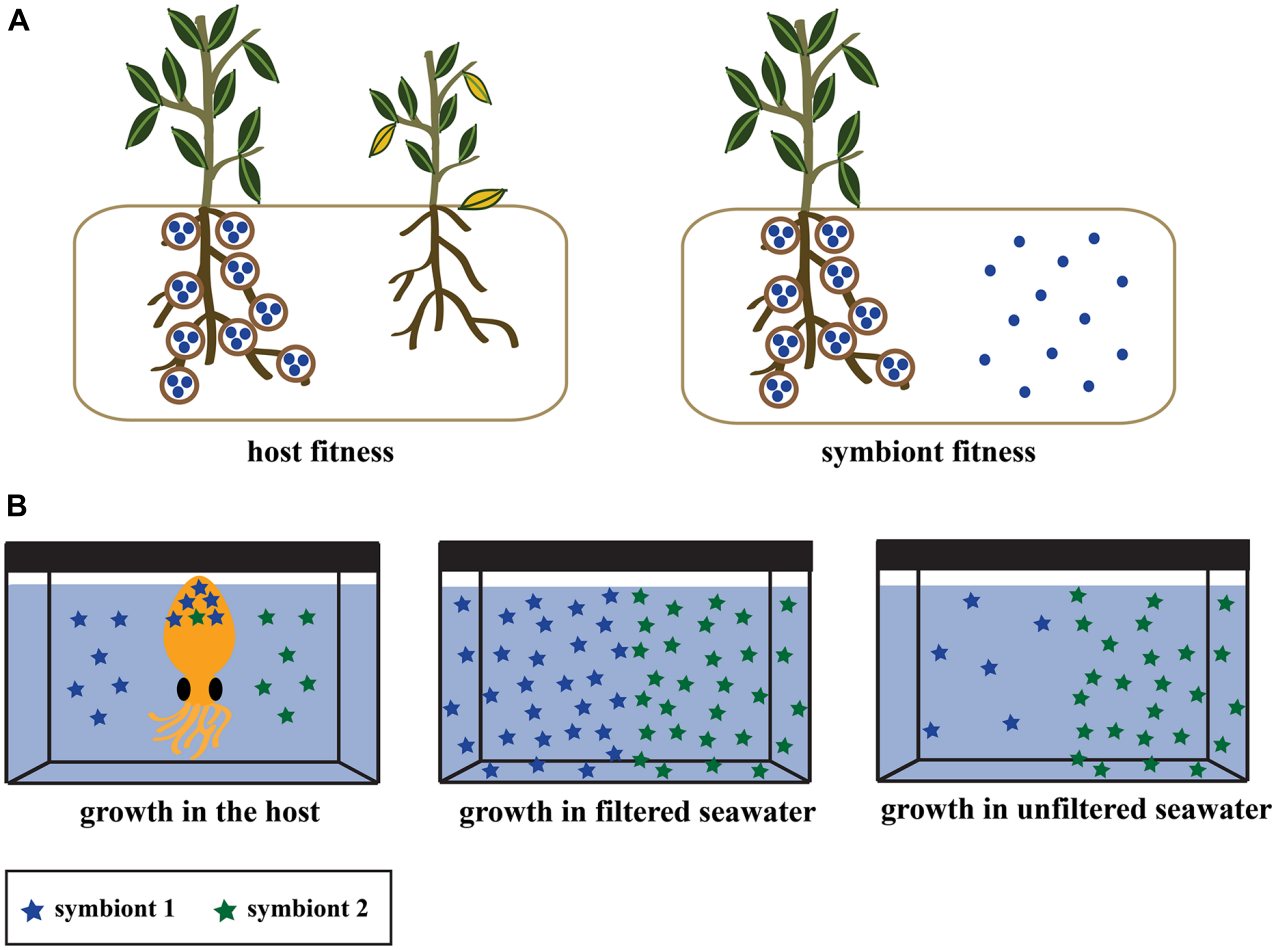
**(A)** Experimental designs to test the effect of symbiosis on host fitness (left) and symbiont fitness (right). Both experiments involve measuring growth or other fitness parameters (see section Recommendations for Investigating Symbiont Fitness) in the presence and absence of their partner. Experiments on host fitness have been performed in diverse systems, but the equivalent symbiont fitness experiment is rarely performed. **(B)** Experimental design from Wollenberg and Ruby (2012) for measuring the relative growth of two groups of bobtail squid symbionts within naturally infected hosts. Competition assays were performed to test within-host fitness by inoculating the seawater of a hatchling squid with a symbiont strain from each symbiont group (left). A separate experiment confirmed that the symbionts had an equal ability to colonize the squid after single-strain inoculations (not pictured). Symbiont growth was tested in the environment by inoculating filtered (middle) and unfiltered (right) seawater from the natural habitat of the squid and symbiont.

Most research of mutualisms has focused on the host, as they are larger and usually a more tractable experimental organism. The effect of microbial association on hosts is routinely tested by comparing fitness in hosts with and without symbionts (**Figure 1A**; e.g., Kikuchi et al., 2007). Analogous experiments for symbionts are rarely performed, even in well-described systems. It is often assumed that symbiont fitness is higher in hosts relative to other niches because they receive a competition-free environment, reduced predation, or host-derived nutrients. Population size is a straightforward way to measure microbial fitness (i.e., the replication capacity of a clonal population), but it should be used to quantify symbiont fitness in the same way that it is for hosts – as the difference in replication in the presence and absence of its interacting partner. When tested, some experiments have shown that symbionts suffer deleterious effects or costs such as suppressed growth in hosts (Ahmadjian, 1993; Wooldridge, 2010; Login and Heddi, 2013; Udvardi and Poole, 2013). The presence of some costs in the host relative to other niches does not necessarily preclude the symbiont from gaining a net fitness benefit through host association [e.g., acquiring genetic diversity through horizontal gene transfer (HGT)], but it does suggest an important aspect that should be considered.

The semantics of symbiosis may be partially to blame for the neglect of microbes. There have been two prominent uses of “symbiosis” over the past century. The first follows from the definition of symbiosis by de Bary as “the living together of unlike organisms” and is applied to interspecies associations regardless of the relationship (parasitism, commensalism, or mutualism; Douglas, 2010; Leigh, 2010). In the second, symbiosis is synonymous with mutualism and indicates a generally beneficial relationship. This is usually applied when it is known that the host benefits from an association and implies that the symbiont does as well. Here we consider any long-term, intimate association to be a “symbiosis” while reserving mutualism for only those interactions known to be beneficial for both partners.

Here we evaluate evidence for reciprocal benefit in presumed mutualistic microbial symbioses, emphasizing environmentally acquired (horizontal) microbial symbionts in eukaryotic hosts. We also re-examine the role of hosts and microbes in symbioses in light of evidence for symbiont benefit. Although it has previously been recognized that symbionts must be more thoroughly investigated (Douglas and Smith, 1989; Bronstein, 2001; Wilkinson and Sherratt, 2001; Kereszt et al., 2011), recent advances in technology and new study systems provide novel tools and opportunities for investigating the symbiont side of symbiosis.

## AN EVALUATION OF ASSUMED SYMBIONT BENEFITS

### COMPETITION

It is assumed that microbial symbionts benefit from a competition-free environment inside hosts because they live in the absence of other microbes that compete for resources. While some systems have monoclonal symbiont populations (Gage, 2002; Martens et al., 2003; Kubota et al., 2007; Dubilier et al., 2008; Aanen et al., 2009), likely due to bottlenecks during repeated vertically transmission or winnowing during horizontal transmission, not all host-symbiont associations are monoclonal. Within-host competition between strains is important for pathogen fitness (Bell et al., 2006) and some vertical symbionts (Oliver et al., 2006). This is likely also true for horizontal symbionts as hosts from many systems harbor multiple symbiont genotypes (Baker and Romanski, 2007; Dubilier et al., 2008; Fay et al., 2009; FitzPatrick et al., 2012; Van Horn et al., 2012; Garcia et al., 2014). Even hosts with strict colonization requirements and entry mechanisms, like bobtail squid which select specific strains of *Vibrio fischeri* from diverse microbes in seawater, contain multiple symbiont genotypes (Wollenberg and Ruby, 2009).

Competition in a polyclonal symbiont population can result in decreased growth for one species or genotype (Elliott et al., 2009; Baker et al., 2013; Engelmoer et al., 2014) or lower symbiont titers (Mouton et al., 2004). Mycorrhizal fungi, for instance, have lower abundance in plant roots when co-inoculated relative to single inoculations. Furthermore, competition between these fungi is stronger within the host compared to the rhizosphere (Engelmoer et al., 2014). Coexistence with other symbionts, however, can be beneficial. Double or triple infections of *Wolbachia* in the wasp *Asobara tabida*, for example, increase the abundance of a specific *Wolbachia* genotype relative to single infections with that genotype only (Mouton et al., 2004). Co-infections, therefore, are a necessary but not sufficient condition for competition and there is no a general framework for predicting the conditions in which co-infections will promote or hinder a symbiont’s fitness. Future research on within-host competition is needed, and should be considered in the context of mechanisms, such as partner choice and sanctioning, that may reduce or prevent polyclonal infections and competition (Bull and Rice, 1991).

### PREDATION AND THE HOST IMMUNE SYSTEM

In non-host environments, microbes are attacked by pathogens and preyed upon by predators such as nematodes, zooplankton, and filter-feeding invertebrates. In hosts, symbionts still face pressures akin to predation. Hosts have potent immune defenses with which both horizontal (Dunn and Weis, 2009) and vertical (Wang et al., 2009; Laughton et al., 2011) symbionts must sometimes contend. These defenses are analogous to predators as they suppress population growth and can eliminate organisms from an environment (Sachs and Wilcox, 2006; Kim et al., 2013). In some cases, a multitude of bacteria enter a host but cannot pass increasingly specific checkpoints to establish within the host (Nyholm and McFall-Ngai, 2004; Kim et al., 2013). Microbes are killed by a range of host immune responses, including phagocytosis, antimicrobial peptides, and reactive oxygen species (Davidson et al., 2004; Login and Heddi, 2013). Hosts can also suppress or regulate established symbiont populations. Carpenter ants reduce bacterial symbiont populations through modulation of an immune response during development (Ratzka et al., 2013). Similarly, tsetse flies express antimicrobial peptides in symbiont-housing cells to regulate symbiont populations (Login et al., 2011). Although it is not known if host control of symbiont growth via immune system “predation” is universal, it is clear that symbionts do not grow unfettered in hosts.

Symbiont growth may also be controlled using mechanisms unconnected to the immune system. Rhizobia root nodule bacteria (Udvardi and Poole, 2013), algal symbionts of corals (Wooldridge, 2010), insect bacterial symbionts (Login and Heddi, 2013), and lichen photobionts (Ahmadjian, 1993) can have lower growth rates relative to their free-living counterparts. The growth of *Symbiodinium* algae is suppressed in corals relative to free-living *Symbiodinium*, but the rate of photosynthesis is comparable in both populations (Muscatine et al., 1984; Falkowski et al., 1993), suggesting algal energy is directed toward producing photosynthate for the host rather than self-growth. In other hosts, proliferating *Symbiodinium* cells are preferentially expelled over non-proliferating cells (Baghdasarian and Muscatine, 2000). However, growth suppression of certain symbiont cells in the host does not single-handedly indicate a deleterious effect on symbionts. The real indicator of a beneficial association is an increased capacity to reproduce in the host relative to the non-host niche, which has not been sufficiently addressed.

### HOST-PROVIDED NUTRITION

There are clear examples in which symbionts receive nutrients like amino acids (Graf and Ruby, 1998; Macdonald et al., 2012) from hosts. Rhizobia bacteria receive numerous compounds from their plant hosts, including amino acids, sugars, and trace ions (Prell et al., 2009; Udvardi and Poole, 2013). However, it is unclear whether any of these nutrients are beneficial to the symbiont. In the case of amino acids, free-living and cultured rhizobia can synthesize branched chain amino acids on their own, but the synthesis of these amino acids is significantly down-regulated in root nodules, and rhizobia in the host rely solely on the plant for these amino acids (Prell et al., 2009). In this state, “symbiotic auxotrophy,” bacteria seem to function more as ammonia-producing organelles rather than organisms seeking to increase their fitness. Similarly, *V. fischeri*, bobtail squid symbionts, receive amino acids, fatty acids and chitin from their hosts (Graf and Ruby, 1998; Jones and Nishiguchi, 2006; Wier et al., 2010). However, there is evidence that *V. fischeri* benefit from these host-derived nutrients or another aspect of host association, as environmental populations are larger in habitats with squid hosts compared to those without squid (Lee and Ruby, 1994; Jones et al., 2007). Ultimately measures of microbial growth along with direct tests of the fate of microbes inside and outside hosts are crucial for understanding the effect of host-derived nutrients.

## RECOMMENDATIONS FOR INVESTIGATING SYMBIONT FITNESS

The effect of microbes on hosts has been quantified in many systems by measuring fitness in symbiotic and aposymbiotic hosts, but the effect of host-association on symbionts has been tested far less frequently (**Figure 1A**). One experiment in the squid-*Vibrio* system serves as a model for symbiont experiments using the comparative fitness approach (**Figure 1B**). Wollenberg and Ruby (2012) inoculated bobtail squid, filtered seawater, and unfiltered seawater with *V. fischeri* strains that were either highly prevalent or rare symbionts in squid hosts. The common symbionts grew as well as the rare symbionts in the squid host and in filtered water, but displayed a distinct population decline in unfiltered seawater (Wollenberg and Ruby, 2012), likely due to predation or competition from other seawater inhabitants. This is one of the only experiments demonstrating that symbionts have an increased reproductive capacity and higher fitness within-hosts relative to non-host environments. It is important to note that this experiment found an effect because it utilized natural environments (ocean water with diverse microorganisms and nutrients) rather than culture based conditions.

Population growth is an appropriate measure of fitness for many microbes because growth and offspring production are usually the same, i.e., binary fission. There are many easy and reliable methods for measuring microbial population growth, including counting by culturing (CFUs or OD_600_), counting labeled cells with a microscope or flow cytometer, and counting gene copies with quantitative polymerase chain reaction (qPCR). However, there are alternative measures of fitness, that include future reproduction (Ratcliff et al., 2012), reproductive structures, e.g., fruiting bodies (Huang et al., 2006), sporulation (Pringle and Taylor, 2002), transmission (Huang et al., 2006), and virulence (Bryner and Rigling, 2012), that can also be employed. These measures are routinely used to measure pathogen fitness; for instance, measuring virulence as a percentage of hosts killed as a proxy for microbial fitness (Parker et al., 2014). These alternative fitness measures may be more appropriate for many symbionts, especially those with complex lifecycles such as fungi (Pringle and Taylor, 2002) and protists (Devreotes, 1989). Certain nodulated rhizobia, for example, undergo multiple rounds of endoreplication, each time doubling the chromosome without completing cell division (Udvardi and Poole, 2013). Therefore, comparing population sizes of rhizobial bacteria in and outside the host using a gene counting method like qPCR would provide an inflated count of population size and an alternative measure would be more appropriate. Additionally, alternative fitness measure may detect a benefit to symbionts even when their relative growth rate is lower in hosts than other niches.

One challenge of comparative fitness assays is duplicating an appropriate non-host environment. For example, gene expression differences between symbiotic and free-living rhizobia have been investigated in many studies, but they have almost exclusively used cell culture as the “free-living” environment (Barnett et al., 2004; Djordjevic, 2004; Capela et al., 2006; Karunakaran et al., 2009; Tatsukami et al., 2013; Peng et al., 2014). Comparison between host-associated and cultured symbionts can provide insight into responses to ecologically relevant conditions, such as low-oxygen and nutrient-limitation, but they cannot duplicate the complexity and heterogeneity of natural conditions. Ideally, fitness experiments would be done in substrate taken directly from the environment, as was the seawater for the *V. fischeri* experiment above. Semi-natural substrates like potting soil or aquarium sea salt mixtures are somewhat more informative than cell culture. In other cases, it may not be known if there is a non-host habitat or what the symbiont’s full habitat range is and coupling symbiosis research with more traditional microbial ecology can inform these experiments (Zahran, 2001; Garcia et al., 2014).

Advances in “omics” technologies (genomics, transcriptomics, etc.) have provided new approaches to investigate symbiont fitness. Although omics approaches do not directly test symbiont fitness, they can illuminate the “terms” of the relationship and hint at benefits. For instance, up-regulation of vitamin production in the host could suggest a nutritional benefit for symbionts, while overexpression of anti-phage proteins may indicate protection of symbionts from pathogens. Omics data can be used to direct and refine comparative fitness assays. For example, simultaneous transcriptome sequencing of *Porites* (a coral) and *Symbiodinium* (its symbiont), revealed that neither partner could synthesize a complete repertoire of amino acids. This, coupled with up-regulation of transport proteins, suggests amino acids are transported between host and symbiont, including amino acids that may be a limiting resource for *Symbiodinium* outside the host (Shinzato et al., 2014). Targeted experiments could test the fitness effect of nitrogen-limitation or removal of specific amino acids on *Symbiodinium* growth in the host and seawater. Omics studies may be especially useful when laboratory fitness assays do not reveal any difference between host-associated and free-living microbes (because the benefit depends on a factor not present in the lab).

One disadvantage of growth as a fitness measure is its emphasis on short-term, immediate benefits at the expense of long-term, rare benefits, which could include access to novel genetic diversity or dispersal. HGT is an important source of novel DNA in prokaryotes, and there is considerable evidence that HGT is important in symbiosis (Marchetti et al., 2010; Husnik et al., 2013; McFall-Ngai et al., 2013). HGT is impeded by separation between appropriate donor-recipients pairs, which could be overcome when closely-related prokaryotes, which are more likely to be compatible (Popa and Dagan, 2011), come together in a host. HGT is particularly prevalent in proteobacteria (Nielsen et al., 2014), phyla rife with insect (Kikuchi et al., 2011), marine invertebrate (Dubilier et al., 2008; Bright and Bulgheresi, 2010), and leguminous plant symbionts (Zahran, 2001). Genomic analysis indicates genes that control host specificity and colonization in the proteobacteria *Xenorhabdus nematophila* (Cowles and Goodrich-Blair, 2008) and *V. fischeri* (Mandel et al., 2009) have likely been acquired via HGT. Although some proteobacterial endosymbionts have lower rates of HGT than their close relatives (Kloesges et al., 2011), this is not true for proteobacteria in mammalian guts (McFall-Ngai et al., 2013). Additionally, HGT may be especially adaptive for horizontal symbionts as they could access novel DNA within-hosts, even if host association was detrimental to short-term fitness. Dispersal may be a similarly rare but beneficial event. Mobile hosts such as flying insects or pelagically dispersed coral larvae (Wirshing et al., 2013) may transport symbionts to novel environments or hosts that better support symbiont growth. Dispersal would be of particular benefit in systems where local extinction is possible. These rare benefits may provide small or hard-to-measure fitness gains to symbionts that outweigh other short-terms costs associated with inhabiting a host or another niche.

Finally, in order to persist, horizontal symbionts must outlive their host by dispersing to a new host or free-living habitat. In some systems, there is clear release of viable symbionts back into the environment. Bobtail squid expel ∼95% of their symbionts in a daily cycle (Lee and Ruby, 1994) and gene expression studies indicate symbionts prepare for life outside the host before expulsion by up-regulating flagellar genes and making metabolic changes (Jones and Nishiguchi, 2006; Wier et al., 2010). Some legumes (Bright and Bulgheresi, 2010) and marine invertebrate hosts (Sachs and Wilcox, 2006), including coral (Baghdasarian and Muscatine, 2000), also release viable symbionts, though this has primarily been considered a way to rid themselves of poor symbionts (Douglas, 2008). In contrast, some hosts can kill, digest, or otherwise prevent viable symbionts from cycling back into the environment. Some rhizobia have undergone such extreme physiological changes that they are no longer viable outside the host, though they do remain metabolically active (Mergaert et al., 2006). In many systems, it is unknown whether symbionts can leave the host much less whether they are viable in the environment. Determining whether a symbiont can leave the symbiosis and proliferate is important as transmission dynamics, the cornerstone of pathogen fitness and evolution (de Roode et al., 2008), undoubtedly play a role in the ecology and evolution of beneficial symbionts as well.

Symbiosis is an important and intensely studied topic in evolution and ecology. However, core concepts including how beneficial symbioses are formed and maintained over evolutionary time are not well developed. The most common hypothesis is that these associations are maintained through mutual benefit. However, in cases where there is no evidence of a symbiont benefit, symbionts may instead be more akin to prisoners or farmed crops than equal partners. Even if symbionts do exhibit increased reproductive ability in hosts, this could ultimately be of little evolutionary benefit, in much the same way cattle populations increase through ranching but, as most cattle are sacrificed prior to reproduction, they do not receive a fitness benefit. Therefore, it is important to determine whether hosts imprison symbionts and whether symbionts have adaptations to evade capture in addition to measuring costs and benefits of presumed mutualisms (Douglas, 2008). Even in this warden-prisoner model of host–microbe association, it is important to recognize there may be both costs and benefits to associating with a host and to identify the short- and long-term fitness consequences for microbes in a variety of contexts. Ultimately, it is clear that progress in symbiosis research requires inclusion of the symbiont side of symbiosis.

## AUTHOR CONTRIBUTIONS

Justine R. Garcia and Nicole M. Gerardo developed the ideas presented here. Justine R. Garcia wrote the manuscript and Nicole M. Gerardo revised and edited it. Justine R. Garcia and Nicole M. Gerardo both approve of the final version of this manuscript and take responsibility for all its contents.

## Conflict of Interest Statement

The authors declare that the research was conducted in the absence of any commercial or financial relationships that could be construed as a potential conflict of interest.
